# Mechanisms of nitrogen deposition effects on temperate forest lichens and trees

**DOI:** 10.1002/ecs2.1717

**Published:** 2017-03-01

**Authors:** Therese S. Carter, Christopher M. Clark, Mark E. Fenn, Sarah Jovan, Steven S. Perakis, Jennifer Riddell, Paul G. Schaberg, Tara L. Greaver, Meredith G. Hastings

**Affiliations:** 1 US Global Change Research Program, ICF Contractor, 1800 G Street NW, Suite 9100, Washington, D.C. 20006 USA; 2 Department of Chemistry, Brown University, 324 Brook Street, Providence, Rhode Island 02912 USA; 3 US EPA, Office of Research and Development, Global Change Research Group, 1200 Pennsylvania Avenue, N. W., Washington, D.C. 20460 USA; 4 USDA Forest Service, Pacific Southwest Research Station, 4955 Canyon Crest Drive, Riverside, California 92507 USA; 5 USDA Forest Service, Pacific Northwest Research Station, 620 SW Main Street, Portland, Oregon 97205 USA; 6 US Geological Survey, Forest and Rangeland Ecosystem Science Center, Corvallis, Oregon 97331 USA; 7 Sustainable Technology Program, Mendocino College, 1000 Hensley Creek Road, Ukiah, California 95482 USA; 8 USDA Forest Service, Northern Research Station, 705 Spear Street S, Burlington, Vermont 05405 USA; 9 National Center for Environmental Assessment, US Environmental Protection Agency, Research Triangle Park, North Carolina 27711 USA; 10 Department of Earth, Environmental, and Planetary Sciences, Institute at Brown for Environment and Society, Brown University, 324 Brook Street, Providence, Rhode Island 02912 USA

**Keywords:** acidification, eutrophication, forests, lichens, mechanism of effect, nitrogen deposition, trees

## Abstract

We review the mechanisms of deleterious nitrogen (N) deposition impacts on temperate forests, with a particular focus on trees and lichens. Elevated anthropogenic N deposition to forests has varied effects on individual organisms depending on characteristics both of the N inputs (form, timing, amount) and of the organisms (ecology, physiology) involved. Improved mechanistic knowledge of these effects can aid in developing robust predictions of how organisms respond to either increases or decreases in N deposition. Rising N levels affect forests in micro- and macroscopic ways from physiological responses at the cellular, tissue, and organism levels to influencing individual species and entire communities and ecosystems. A synthesis of these processes forms the basis for the overarching themes of this paper, which focuses on N effects at different levels of biological organization in temperate forests. For lichens, the mechanisms of direct effects of N are relatively well known at cellular, organismal, and community levels, though interactions of N with other stressors merit further research. For trees, effects of N deposition are better understood for N as an acidifying agent than as a nutrient; in both cases, the impacts can reflect direct effects on short time scales and indirect effects mediated through long-term soil and belowground changes. There are many gaps on fundamental N use and cycling in ecosystems, and we highlight the most critical gaps for understanding potential deleterious effects of N deposition. For lichens, these gaps include both how N affects specific metabolic pathways and how N is metabolized. For trees, these gaps include understanding the direct effects of N deposition onto forest canopies, the sensitivity of different tree species and mycorrhizal symbionts to N, the influence of soil properties, and the reversibility of N and acidification effects on plants and soils. Continued study of how these N response mechanisms interact with one another, and with other dimensions of global change, remains essential for predicting ongoing changes in lichen and tree populations across North American temperate forests.

## Introduction

Historically, nitrogen (N) supply to most terrestrial and aquatic ecosystems was low relative to organismal demands, but anthropogenic influences on the N cycle have greatly elevated reactive N (Nr) inputs from atmospheric deposition over pre-industrial levels. During the last half century, the production of Nr by humans has outstripped that by the natural world (Galloway et al. 2003). From 1860 to the early 1990s, land-use change decreased the natural terrestrial production of N by 15% (from 120 Tg N/yr to ~107 Tg N/yr), while anthropogenic production of Nr increased tenfold (from ~15 Tg N/yr to ~156 Tg N/yr; Galloway et al. 2004). Much of this additional Nr (in both reduced and oxidized forms) has accumulated in the environment as natural processes for converting Nr back to unreactive N_2_ gas were unable to keep pace with human production of Nr (Galloway et al. 2003).

The accumulation of Nr from anthropogenic sources impacts both ecosystems and humans (Compton et al. 2011), with disproportionately large impacts because one atom of Nr can cascade through the environment in different molecular forms, potentially affecting the atmosphere, terrestrial, aquatic, and estuarine ecosystems, and human health (Galloway et al. 2003). Reactive N is often limiting in terrestrial systems, and this N scarcity matters for Nr responses. In fact, terrestrial organisms and communities are often adapted to low N and have responded to this via a diversity of N acquisition strategies, including the efficient retention and recycling of N among biota and new, but often limited, N inputs from fixation. While many effects of N on aquatic systems result from non-point sources of N (e.g., from fertilizers), N deposition to landscapes can also have a variety of effects on terrestrial systems, including nutrient imbalances in leaf tissues (Schaberg et al. 2002), changes in plant community composition (Stevens et al. 2010, Simkin et al. 2016), and soil acidification (McNulty et al. 2007).

Trees, the dominant life form in forests, can also be sensitive to excess N, whether alone, or in concert with other air pollutants such as acidic sulfur (S) deposition and ozone (O_3_) exposure. A recent study found that of the 24 dominant species across a 19-state area in the northeastern United States (U.S.), three showed a net negative growth response to N deposition and eight showed a reduced probability of survival (Thomas et al. 2010). Trees are also inextricably linked to the health of another sensitive life form, lichens, by serving as the substrate on which they grow and by altering local N deposition via capture of aerosols, gases, cloudwater, and precipitation. Lichens and trees also provide a variety of ecosystem services to society. Epiphytic lichens are an important source for nesting material for many species of birds; they provide key winter forage for species such as caribou, rabbit, and vole; and they are used by various native cultures for medicinal properties and are increasingly being studied by Western medicine for their pharmacological properties (Brodo et al. 2001, Ranković 2015). Forest trees also provide habitat for many species of game and non-game animals, substrate for nesting material of many bird species, timber for firewood and commercial production, carbon (C) sequestration, climate and water regulation, as well as various medicinal uses by traditional cultures (MEA 2005, USDA FEIS 2016).

To better understand the impacts of increased N deposition on temperate forests, this paper reviews the underlying mechanisms at the cellular, individual, and community or ecosystem levels of organization, which determine how N deposition affects species within forested ecosystems. We focus here on temperate forests, and while some of the mechanisms may be general, tropical (Huang et al. 2015, Chen et al. 2016, Li et al. 2016a) and boreal (Fleischer et al. 2015, Binkley and Hogberg 2016) forests are not covered here. Throughout, the degree of observed response to N will be a function of N status, the status of other nutrients (e.g., P, Ca), and other ecosystem factors such as stand age and composition. We focus on trees and on lichens that live on trees (epiphytes) and rocks (epiliths); though, some of these mechanisms are relevant to other forest life forms such as the understory herbaceous or shrub layer and, to a lesser extent, epiphytic vines and herbs. Many of these mechanisms and responses also apply to bryophytes (e.g., mosses and liverworts) such as competition for light (Van der Wal et al. 2005), and we refer the reader elsewhere for syntheses of N cycling and deposition effects on bryophytes (Soares and Pearson 1997, Turetsky 2003). Our focus on epiphytic and epilithic lichens reflects their utility as bioindicators of air quality in forests; bryophytes can also be used as bioindicators (Mitchell et al. 2004). Lichens are particularly sensitive to air quality because they have no roots and take up water, solutes, and gases over their entire thallus surface (unlike other epiphytic plants), so they subsist almost entirely on atmospheric moisture and nutrients (Hauck 2010, Jovan et al. 2012). These lichens are more likely to suffer certain acidification effects than higher plants because the plant leaf cortex and endogenous polyamines (organic compounds that contain at least two amino acids) may act as a barrier to the direct effects of acidic deposition (Pirintsos et al. 2009) unlike in lichens. Our focus on trees reflects their dominant role as the defining structural and functional attributes of forests, and in connections to other ecosystems and global processes that regulate biodiversity, climate, and other ecosystem services (Fenn et al. 1998, MEA 2005).

The N cycle to varying degrees also shapes cycles of other essential macronutrients like phosphorus (P), calcium (Ca), and magnesium (Mg), so increasing N can affect other cycles (Perakis et al. 2006, Vitousek et al. 2010). Furthermore, natural systems vary in their ambient N availability, which contextualizes their responses to Nr, and when Nr is added to systems with high N, more immediate negative effects may be observed (Fenn et al. 1998). When discussing the effects of N on ecosystems, it is also important to keep in mind the ways in which other pollutants such as S and O_3_ deposition, and other environmental stressors like climate change, may affect similar systems or act synergistically with N on already-stressed plants. Ozone is a widespread phytotoxic pollutant and contributes to widespread harm in U.S. forests (Karnosky et al. 2007). While sulfur dioxide (SO_2_) is not a major pollutant affecting U.S. forests as a direct toxicant, sulfate (SO_4_^2−^) deposition does greatly contribute along with N deposition to acidification. Accumulated SO_4_^2−^ in soils is a legacy of much higher S deposition in past decades and continues to affect soils and watersheds in certain areas of the eastern United States (Rice et al. 2014 and references therein). When considering the effects of chronic N deposition, integration of responses to multiple factors is needed because ecosystems respond to the totality of environmental stressor exposure rather than to just individual factors.

Nitrogen is an essential biological macronutrient, and N limitation in terrestrial ecosystems broadly constrains primary production (LeBauer and Treseder 2008). Accordingly, increases in N availability typically increase the productivity and growth of some tree species, with cascading effects that often increase total forest C sequestration. Recent syntheses of N effects on boreal and temperate sites indicate aboveground stimulation ranging from 25 to 61 kg C/kg N (Thomas et al. 2010, Butterbach-Bahl et al. 2011, Pinder et al. 2013), with broad evidence for increases in soil C storage in organic horizons, although mineral soil changes are more difficult to detect (Liu and Greaver 2010).

While increased N deposition in forests stimulates growth of some species, N availability in excess of biological needs has also been associated with reduced productivity and increased mortality (e.g., Aber et al. 1998). It is this latter circumstance of negative consequences that is the focus of our review.

## Overview of N Mechanisms Affecting Trees and Lichen

The mechanisms by which N deposition affects lichens and trees display both similarities and differences. Bobbink et al. (2010) identified four main mechanisms through which N deposition affects terrestrial ecosystems: direct toxicity, changes in plant-species interactions because of more bioavailable N compounds and eutrophication, soil-mediated impacts of acidification, and increased susceptibility to secondary stressors. Competitive interactions between and among lichen, herb, and tree species also play a role in biodiversity loss—mediated by increasing biomass of nitrophilic species that leads to shading and light competition (Fenn et al. 2003a, Hauck and Wirth 2010, Johansson et al. 2012, Munzi et al. 2014).

**Fig. 1.**
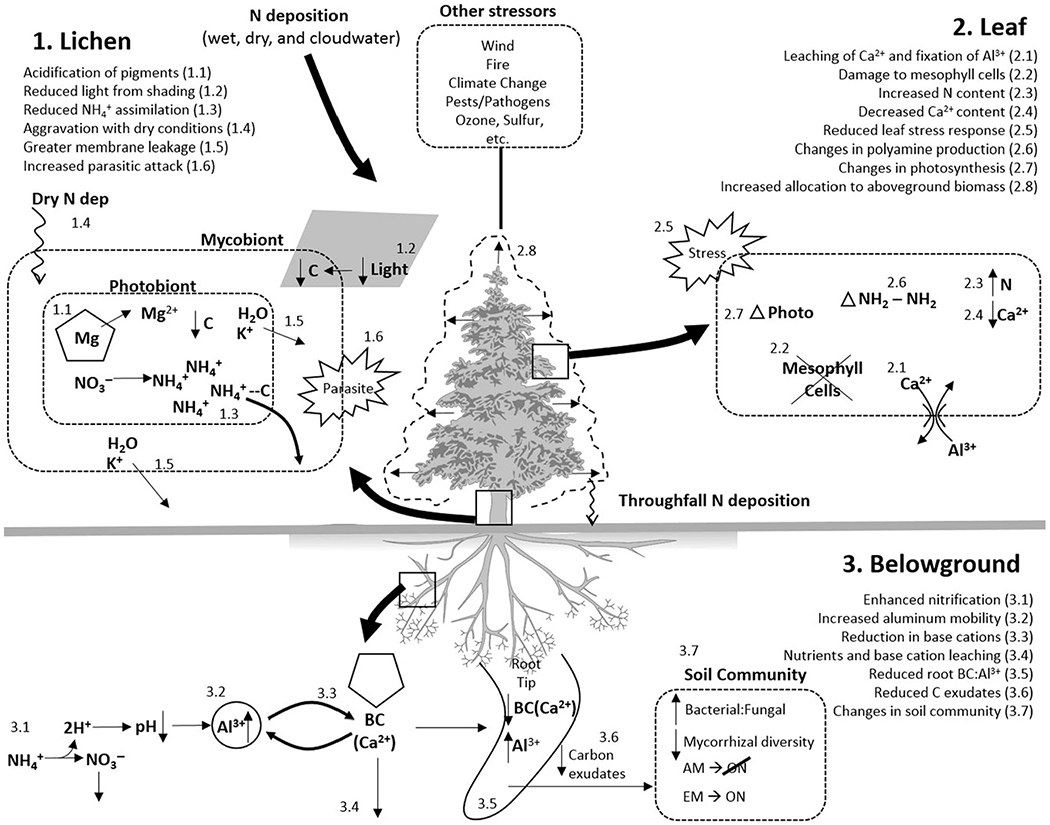
Conceptual diagram of the effects of nitrogen deposition on epiphytic lichens and trees. Major areas of effect are separated by straight lines clockwise for lichen (1.1–1.6), for leaf and aboveground tissues (2.1–2.8), and for belowground tissues and processes (3.1–3.7). For lichen, included in the diagram are a decline in the number of carbon skeletons from acidification of photosynthetic pigments (1.1) and reduced light levels from nearby plant growth (1.2), a fewer number of carbon skeletons reducing the ability to assimilate NH_4_^+^ leading to an accumulation of this toxic compound (1.3), the magnified effect under drier conditions (1.4), greater membrane leakiness to K of the photobiont and mycobiont (1.5), and possible parasitic attack (1.6). For the leaves and above-ground tissue, the diagram shows foliar leaching of Ca and fixation of A1 to the cell wall and/or cell membrane (2.1), subsequent damage to mesophyll cells (2.2), increases in foliar N content (2.3), decreases in foliar Ca content (2.4), reductions in the leaf-level stress response (2.5), changes in the production of polyamines (2.6), increases or decreases in photosynthesis (2.7), and increases in allocation to aboveground biomass (2.8). In the soil is shown acidification through enhanced nitrification (3.1), increased aluminum mobility (3.2), reduction in base cations on the soil exchange sites (pentagon, 3.3), and subsequent loss via leaching of nutrients and base cations (3.4). All of this along with other factors can lead to reductions in the BC:A1 ratio in the root (3.5) and reductions in carbon-rich root exudates (3.6) which can affect soil microbial communities, especially for arbuscular mycorrhizal fungi (AM)-dominated systems that cannot access complex organic N (3.7). Also listed but not detailed are several other stress factors that can modify the response to trees and/or lichen. Several processes are not included, including modifying factors from P, possible reductions in belowground biomass, and other factors mentioned in the text.

The central mechanism affecting lichens is direct toxicity from Nr gases and aerosols (both oxidized and reduced forms of Nr, explained in more detail below) and, to a lesser extent, changes in species interactions. Because lichens consist of symbiotic relationships between fungi and algae or cyanobacteria, it is also important to determine how the symbiotic partners respond differently to increasing N deposition. Cyanobacteria, sometimes called blue-green algae, are prokaryotic organisms, while green algae are eukaryotic, potentially leading to different mechanisms of effect for the symbiotic partners (see Fig. 1 and “Lichen” sections for more detail).

The primary mechanisms through which N deposition affects trees are mostly soil-mediated; though, some evidence suggests that aerial impacts, secondary factors, and competition also contribute (Bobbink et al. 2010, Thomas et al. 2010). Such mechanisms can include acidification effects like base cation depletion and aluminum (Al) toxicity, or eutrophication effects, or both. Importantly, soils can display wide natural variation in background N availability and buffering capacity, even in relatively small geographic areas (Hynicka et al. 2016) that determine which mechanisms of effects may occur (Clark et al. 2007). Soils also differ among regions, wherein regional differences in water balance or underlying geology modulate the extent of soil acidification (Fenn et al. 1998, Grulke et al. 1998). Increased susceptibility to secondary stressors (e.g., low or high temperatures, low moisture availability, and insect herbivory), and changes in ratios between above- and belowground net primary production are other important mechanisms through which N deposition can alter tree health and productivity (Grulke et al. 1998, Fenn et al. 2003a, b, see Fig. 1 and “Tree” sections for more information).

## Mechanism of Effect from Atmospheric Deposition on Lichens

### Background

Because lichens consist of symbiotic relationships between fungi and algae or cyanobacteria, it is important to determine how the individual symbiotic partners, and their joint life form, respond to increasing N deposition. In the Mechanism of Effect section below, we review recent literature detailing the ways in which N deposition directly and indirectly affects lichen species, and in the Research Gaps section, we have identified areas in which further work could improve our understanding of mechanisms of effects from the cell to the ecosystem.

### Mechanism of effect

#### At the tissue and cellular level—physiological responses.—

Under natural conditions, lichens show a high efficiency for simultaneous uptake of both oxidized and reduced N species. However, when lichen species were exposed to N concentrations in excess of their needs, reduced N was taken up more efficiently than oxidized species (Hauck 2010). Thus, it has been argued that reduced N is a more important N source than oxidized species (Hogan et al. 2010a). While it is indeed likely that lichens respond to multiple forms of Nr, Hogan et al. (2010a) reported that lichens take up ammonium (NH_4_^+^) more quickly than oxidized forms, which indicates that the responses of lichens to Nr pollution may depend on the relative abundance of oxidized and reduced Nr species (Hogan et al. 2010a and references therein). Multiple studies have also shown that lichens are very sensitive to NH_4_^+^ concentrations, which can affect their ability to assimilate C, and that the proportion of atmospheric deposition as reduced N in the United States is increasing (Du et al. 2014, Li et al. 2016b).

At the tissue and cellular level, it is commonly thought that the ability of lichens to tolerate high Nr depends on whether they can provide enough C skeletons to quickly convert NH_4_^+^ into amino acids, thus avoiding the buildup of toxic amounts of free NH_4_^+^ but incurring a metabolic cost (Hauck 2010; Fig. 1.1.3). Consistent with this idea, Hauck and Wirth (2010) showed that shade-adapted lichens, which are mainly found in N-poor environments, respond poorly to eutrophication (Hauck and Wirth 2010). They suggest that low light leads to lower photosynthetic production of C skeletons critical for efficient N assimilation (Fig. 1.1.2).

Depending on the species (Johansson et al. 2011, Paoli et al. 2015) and certain climate factors, the acidification of pigments from increasing N deposition (in the form of NH_4_^+^, NO_3_^−^, and organic N) can harm lichens’ photosynthetic abilities and therefore their ability to capture energy and C (Fig. 1). The acidification of pigments, which also occurs with exposure to SO_2_ removes the central Mg ion from chlorophyll molecules (green pigments), creating phaeophytin (non-photosynthetic) pigments (Fig. 1.1.1). For example, in a study by Riddell et al. (2008), lichen thalli were fumigated with nitric acid, and an increase in the percentage of phaeophytin pigments was observed from 0% to 65% (wet conditions) or 100% (dry conditions). A consequence of this loss of photosynthetic ability is that lichens lose the capacity to photosynthesize or fix carbon dioxide (CO_2_) into energy-storing sugars, restricting the ability of both symbiotic partners to carry out basic metabolic processes like cell repair, growth, and reproduction (Riddell et al. 2008). This loss of photosynthetic ability thus limits the amount of C skeletons available to assimilate NH_4_^+^ into other metabolic compounds like proteins. Lichens with cyanobacteria as the photobiont may be more susceptible to these effects of acidification (e.g., decreases in both N fixation and photosynthetic ability). This is because their reproduction, which includes both the germination of ascospores and the abundance and the dispersion of free-living cyanobacteria, is more acid sensitive than green algae (Hallingback and Kellner 1992).

Phaeophytin production increased more rapidly under dry conditions than under more humid ones, suggesting that climate may exacerbate acidification effects (Riddell et al. 2008). Lichens in humidified chambers had slower declines in their C exchange capacity (net photosynthesis and dark respiration) and fewer declines in their chlorophyll content (Riddell et al. 2008; Fig. 1.1.4). If chlorophyll-bearing cells in plants were acidified, similar results would likely be seen (Riddell et al. 2008). However, this is less likely to happen to higher plants than to lichens because the plant leaf cortex and endogenous polyamines may act as a barrier to the direct effects of acidic deposition (Pirintsos et al. 2009). Humidity may have a mitigating influence on lichens’ abilities to tolerate excess N by allowing the lichen to repair some of the cellular damage during the treatment period (Riddell et al. 2008). Potassium (K) ion loss is one indicator of how lichens respond to additional Nr. Increased losses of K^+^ were observed with longer exposure and greater dosage of Nr (Riddell et al. 2008; Fig. 1.1.5 and 1.1.6).

Phosphorous limitation is another possible mechanism for the negative effects of excess N on lichens. Phosphorus limitation is often depletion-driven, which means that due to low P inputs, cumulative P losses over time (e.g., erosion) contribute to a terminal steady state characterized by significant P depletion and limitation (Vitousek et al. 2010). In an N addition experiment, the co-addition of P (using sprayers) enabled lichens characteristic of nutrient-poor habitats to tolerate heavy N loads (Pilkington et al. 2007). The more recent work of Johansson et al. (2011) suggested P additions may mitigate or intensify the negative effects of N depending on species, symbiont, and their N:P ratio.

In simulated N deposition studies (Hogan et al. 2010a, Johansson et al. 2010) and N gradient studies (Hogan et al. 2010b), increasing N was associated with increasing N:P ratios in lichens, suggesting excess N-induced P limitation. While the physiological consequences of P limitation in lichens have not been explored to the same degree as vascular plants, a general hypothesis is that sufficient P is needed for lichens to invest additional N in new photobiont cells (Johansson et al. 2011), thus increasing photosynthetic capacity and the provision of C skeletons needed to fix excess N (Palmqvist and Dahlman 2006). Increasing P supply has been shown to greatly increase lichen productivity in some cases. For instance, one study reported a doubling of annual biomass in the cyanolichen *Lobaria pulmonaria* after just one application of P (McCune and Caldwell 2009), with additional work demonstrating that responses to P varied with background levels (Marks et al. 2015). Benner and Vitousek (2007) observed a “bloom” in canopy lichens, including both cyanolichen and green algal species, in tropical forests where soil was fertilized by P. In high N environments, studies have shown increased investment in P acquisition in both plant and lichen systems via increased activity of the enzyme phosphomonoesterase (Hogan et al. 2010a, b).

#### At the individual level.—

In N bioindication studies that use lichen communities to estimate N deposition, two or three groups of lichen indicator species are commonly used: oligotrophs (i.e., acidophytes; highly N sensitive), eutrophs (i.e., nitrophytes; highly N tolerant or “N-loving”), and mesotrophs (species of intermediate sensitivity; Jovan 2008, Geiser et al. 2010). Research suggests that oligotrophs have limited ability to compensate for increased NH_4_^+^ levels with more C assimilation, while eutrophic lichens may actually increase their photosynthetic ability at Nr levels where oligotrophs cannot survive (Gaio-Oliveira et al. 2005, Hauck 2010). Additionally, it has been proposed that eutrophs have a lower cation exchange capacity, an “avoidance” mechanism that helps prevent Nr uptake (Gaio-Oliveira et al. 2005).

Added Nr can also change the ratio between symbiotic partners in lichens, disrupting the symbiotic relationship, causing cellular membrane damage, and increasing the potential for parasitic fungal attacks—all of which lead to reductions in the stability of lichen thalli (Fig. 1.1.7). Based on observations of diminishing photosynthetic ability, decreasing respiration, and cellular membrane damage detectable through K efflux, multiple studies have shown that acidic deposition may harm both the photobiont and the fungus (Riddell et al. 2008, Munzi et al. 2009). However, studies also show species-specific responses in which the symbiotic partners respond differently to altered nutrient supplies, leading to imbalances in their relative biomass (Gaio-Oliveira et al. 2005). With increasing N deposition, lichens may invest more N for photosynthesis, likely in order to replace damaged pigments and other tissues. This can potentially explain the improved growth of the photobiont over the mycobiont (Johansson et al. 2012), which reduces the stability of lichens because the mycobiont is the symbiotic partner responsible for the structure and stability of the thallus (Johansson et al. 2011, 2012).

The intensification of parasitic fungal infections could be another factor in the decline of lichens in some instances (Fig. 1.1.7). One study found that at least one species of parasitic fungus increased with the addition of large applications (25 and 50 kg N/ha) of N (Ström 2011, Johansson et al. 2012). This parasite destroys the cortex of the host and exposes the medulla. During extreme weather events like heavy storms, these parasites, in combination with imbalances in lichens’ symbiotic relationships, could make lichens more susceptible to structural damage (Johansson et al. 2012).

Most work supports the claim that Nr toxicity to lichens mechanisms, and not competitive interactions among lichen, drives the observed losses in lichen biodiversity with added Nr—at least on the shorter time scales that studies typically cover (Johansson et al. 2012). The capacity to handle higher levels of Nr does allow specific lichen species like *Xanthoria parietina* and other eutrophic species to live unaffected by competition from oligotrophs in certain ecosystems. Thus, additional N does have an indirect positive effect on some lichens like *X. parietina*, shielding them from competition from more sensitive species (Munzi et al. 2014).

#### At the community and ecosystem level.—

Research suggests that competitive interactions between lichens and vascular plants may stress lichens by limiting their access to light needed for photosynthesis (Johansson et al. 2012 and references therein; Fig. 1.1.2). While lichens are inherently shade-tolerant with trees and higher-level plants often providing that shade, increased vascular plant biomass in response to N is altering these dynamics. This mechanism of light limitation after eutrophication has been seen within herbaceous communities—where increased plant biomass decreases plant biodiversity because shorter plants do not receive adequate sunlight (Bret-Harte et al. 2008, Hautier et al. 2009, Johansson et al. 2012). Ground-dwelling lichens may be easily outcompeted by vascular plants in dense vegetation, while shade-adapted lichen communities on bark, wood, and rock are most sensitive to eutrophication (Hauck and Wirth 2010). Because the ability to assimilate NH_4_^+^ depends on the availability of C skeletons, it is to be expected that shade-adapted lichens are more vulnerable to eutrophication since their photosynthetic capacity, and thus production of C skeletons, is limited (Hauck 2010).

#### Interactions with other factors—

As mentioned, lichens may also respond to types of Nr—wet vs. dry deposition and oxidized vs. reduced—in different ways; although there is stronger evidence for the differing effects of wet vs. dry deposition than for oxidized vs. reduced forms, an area requires more research. A study in southern California by Jovan et al. (2012) suggests that total N deposition measured in canopy throughfall is the best predictor of eutroph abundance, indicating that eutrophic species respond to many forms of N. A study by Giordani and Malaspina (2016) supports this hypothesis as well. Several studies taken together suggest that community changes might be more drastic if Nr is added as dry deposition of NH_4_^+^, as gaseous HNO_3_, or as gaseous NH_3_ than as wet deposition (Sheppard et al. 2011, Johansson et al. 2012), in agreement with the humidity-regulating vulnerability mechanism discussed above and below. Dry deposition accumulates on thallus surfaces and can then be very concentrated when precipitation occurs. However, this is dependent on the location and event as fog and smog can have very high concentrations as well. In addition, Pearce and van der Wal (2008) show that the concentration and not just the amount of wet atmospheric N deposition can strongly impact moss, and this factor should be evaluated for its applicability to lichens as well.

Precipitation and humidity levels affect how lichens respond to elevated N deposition. Increased precipitation has been shown to increase the critical load of Nr that lichens can tolerate presumably because precipitation dilutes or leaches depositional Nr (Geiser et al. 2010; Fig. 1.1.4). Additionally, moisture may be protective because it allows the lichen to stay physiologically active, allowing them to fix N into less harmful forms to prevent their accumulation. Temperature could be important in similar ways as it affects wetting and drying processes as well as metabolic rates. There is also evidence that at the same N pollution levels, lichen communities in the driest climates tend to be better adapted to high nutrient concentrations than those in wetter climates (Root et al. 2015). In particular, when N concentrations were at background levels, lichen communities tended to be more eutrophic in water-stressed areas (Root et al. 2015). Highly N-sensitive, N-fixing lichens are among the most drought sensitive.

Frahm (2013) offered a possible mechanism for the theory that eutrophic lichens are more drought-tolerant, which would also help to explain why more eutrophs than oligotrophs are found in dry climates. They posited that drought exacerbates lichen sensitivity to N (and vice versa) because deposited N acts as a salt in dry environments, drawing moisture out of lichen cells. Eutrophs may be more drought and N resistant than oligotrophs, in part because their cells have higher osmotic potential (Frahm 2013). The conductivity of eutrophic lichens is higher than oligotrophs, and so the higher osmotic pressure permits the intake of water vapor at lower levels of relative humidity (Frahm 2013). This author also suggested that it is this osmotic tolerance against the salt effects of N compounds, instead of the ability to metabolize, assimilate, and detoxify larger quantities of N that enables eutrophic lichens to be more N tolerant. This is advantageous because it allows eutrophs to survive in dry habitats where those species with lower osmotic pressures cannot.

These insights suggest the need to better understand the impact of N concentration vs. total N loading. For instance, it has been proposed that in the arid forests of southern California, dry N deposition builds up on the surface of lichens all summer and then dissolves in incoming water during pulses of low-volume rain events at the end of the season. This results in a damaging, high concentration of N that then is passed through the thallus (Jovan et al. 2012). Pearce and van der Wal (2008) also suggest the need to differentiate between dose and concentration of atmospheric wet N deposition as it affects plants. While the study by Pierce and Van der Wal is based on work with plants, the issue also likely applies to lichens, especially considering they receive all their N from the atmosphere.

### Research gaps—lichens

Although much progress has been made in recent years, many gaps in our scientific understanding of how lichens respond to increasing N deposition remain, and here we focus on gaps that are critical for understanding potential deleterious effects of N deposition (summarized in Table 1). Many questions remain regarding the mechanisms behind lichens’ tolerance of increasing loads because Nr as a macronutrient is essential and thus naturally occurs at fairly high concentrations in the thallus (Hauck and Wirth 2010, and references therein). The molecular mechanisms controlling how lichens metabolize Nr are not yet well understood, in contrast with the extensive literature available for vascular plants and free-living algae (Hauck 2010). Lessons can be learned from work done on vascular plants, but results from ongoing DNA sequencing of the lichen-forming fungus *X. parietina* are needed before these lessons can be fully integrated with lichen work (Hauck 2010). Genomic analysis can provide insights into the genetic basis of the symbiosis that forms lichens, adaptation to harsh environments, secondary metabolism, and the control of growth rate (Dyer et al. 2006).

Other hypotheses have been suggested to explain connections between the metabolism and physiology of lichens and their tolerance or sensitivity to Nr. Preliminary work has shown that mannitol may be important for N assimilation in at least some lichen species, but more work (e.g., involving the isotopic labeling of reaction intermediates) is needed (Gaio-Oliveira et al. 2005). Another area that needs more exploration is the role of apothecia as N and C sinks in lichen species with a tolerance for N (Gaio-Oliveira et al. 2005). Like in vascular plants, polyamines play a protective role in lichens’ tolerance of N, but the protective mechanism is unknown (Hauck 2010).

Little is known about whether direct NO_3_^−^ uptake differs among species of the photobiont of lichens and about the NH_4_^+^ uptake behavior of photobiont cells living within the lichen thallus (Hauck 2010). Previous studies have indicated that the mycobiont is more affected than the photobiont when exposed to excess Nr. The mechanism for this is not understood, but the biomass of the fungus dominates in the lichen thallus, so the greater sensitivity of the mycobiont could be simply because it has more surface area exposed to the additional Nr (Gaio-Oliveira et al. 2005, and references therein). Phosphorus may also play a role as Johansson et al. (2011) showed that the mycobiont proportion in the thalli decreased more when both N and P were added than when only N was added, and responded negatively when P alone was added.

While the transformation of NH_4_^+^ to NO_3_^−^ is also observed in some vascular plants tolerant to excess NH_4_^+^ supply, the mechanism has not yet been elucidated for lichens (Gaio-Oliveira et al. 2005, and references therein). Understanding how NH_4_^+^ and pH separately affect the ability of lichens to handle increasing Nr loads is an important research question. The effects of both may impact the acidity that the lichen experiences and, when acting in concert, may reinforce each other. However, the two arise from different sources and may have different mechanisms of effect.

More research is also needed to further elucidate the relationship between eutrophication and shaded environments (Hauck 2010) and humidity. If increasing Nr leads to more plant and biomass growth, it might aggravate competition for light and space, potentially causing slow-growing species of lichens and plants to be out-competed (Johansson et al. 2012, and references therein). Cyanolichens also tend to need more water than green algal lichens to start metabolizing, so it would also be insightful to explore how light and humidity interact to affect different lichen types. To better understand and differentiate mechanisms of action, experiments are needed along light and climate gradients to separate the influences of Nr and other factors.

A study in the Los Angeles (California) Air Basin hypothesized that the loss of habitat and lower humidity levels in inland areas driven by urbanization coupled with increased fire incidence may be important factors in the decreases observed in lichen biodiversity, so more work on this interaction is needed (Riddell et al. 2008). Similarly, Fenn et al. (2003a) showed that increased N deposition over time leads to greater fire frequency in certain areas and habitat changes for already-threatened species. Synergies with O_3_ (Riddell et al. 2008) and SO_2_ near point sources (Ra et al. 2005) could also be confounding factors.

Research gaps remain in our collective understanding of the mechanisms through which N deposition affects lichens. Table 1 summarizes these research gaps and more beyond the narrative text provided here. As our analytical techniques improve, researchers will be better equipped to investigate the complex interactions that influence how lichens deal with excess N both physiologically and within their ecosystems.

## Mechanism of Effect from Atmospheric Deposition on Trees

### Background

Nitrogen deposition affects trees in multiple ways that often occur over a longer time frame than for lichens, and these effects—both as the accumulation of additional N (eutrophication) and as acidification—occur aboveground and belowground. For trees, N is commonly the limiting nutrient for primary production and added Nr often stimulates growth (LeBauer and Treseder 2008). However, added Nr can become detrimental to trees particularly at levels that exceed ecosystem sinks (Aber et al. 1998). Most of the mechanisms of N effects on vegetation identified by Bobbink et al. (2010) apply to trees, including direct damage, increased susceptibility to secondary stressors, and long-term negative effects of reduced Nr (i.e., NH_X_; Stevens et al. 2011, Van den Berg et al. 2016). It is likely that N deposition also affects competition among tree species, potentially altering forest composition; though, field evidence is scarce. Soils are the largest N reservoir in terrestrial ecosystems and are the dominant immediate source of tree N uptake—via roots and associated mycorrhizae. Soils can modulate the effects of N deposition on trees and forests, and this in turn may depend on how persistently excess N causes soil acidification and declines in essential base cations (e.g., Ca, Mg) that are required for tree health and productivity (Aber et al. 1998, Hawley et al. 2006, Schaberg et al. 2006, Pardo et al. 2011).

A majority of foliar atmospheric N uptake and assimilation in trees seems to be the uptake of N gases (NH_3_, HNO_3_, and NO_2_) mainly by stomata, although HNO_3_ is also taken up transcuticularly (Bytnerowicz et al. 1999, Sparks 2009). Roughly 20–40% of inorganic atmospheric N is often retained in forest canopies and not collected in throughfall (Friedland et al. 1991, Lovett and Lindberg 1993, Fenn and Bytnerowicz 1997; Fig. 1.2.3); though, not all of that N is assimilated internally through foliage and bark. Much of the retained N may be converted to organic forms or assimilated by other canopy organisms (Rennenberg and Gessler 1999). However, ^15^N label studies in the field indicate that some N uptake occurs through the bark during wet periods (Dail et al. 2009), and the review by Sparks (2009) provides in-depth analyses of the many mechanisms controlling these different forms of uptake and assimilation.

### Mechanism of effect

#### At the tissue and cellular level—physiological responses.—

Much of what happens in above-ground portions of trees originates from changes in the chemistry and ecology of soils and the roots that reside therein. Because it is easier to measure aboveground than belowground, N-associated changes in aboveground physiology are more studied, but throughout this section, it is essential to keep in mind that changes in soil chemistry and function serve as an important precursor to other mechanistic changes in foliage and other aboveground portions of trees. For example, laboratory and field studies have shown that atmospheric N taken up by the canopy is transported to the phloem of roots, which can cause a decline in root N uptake (Rennenberg and Gessler 1999).

**Fig. 2.**
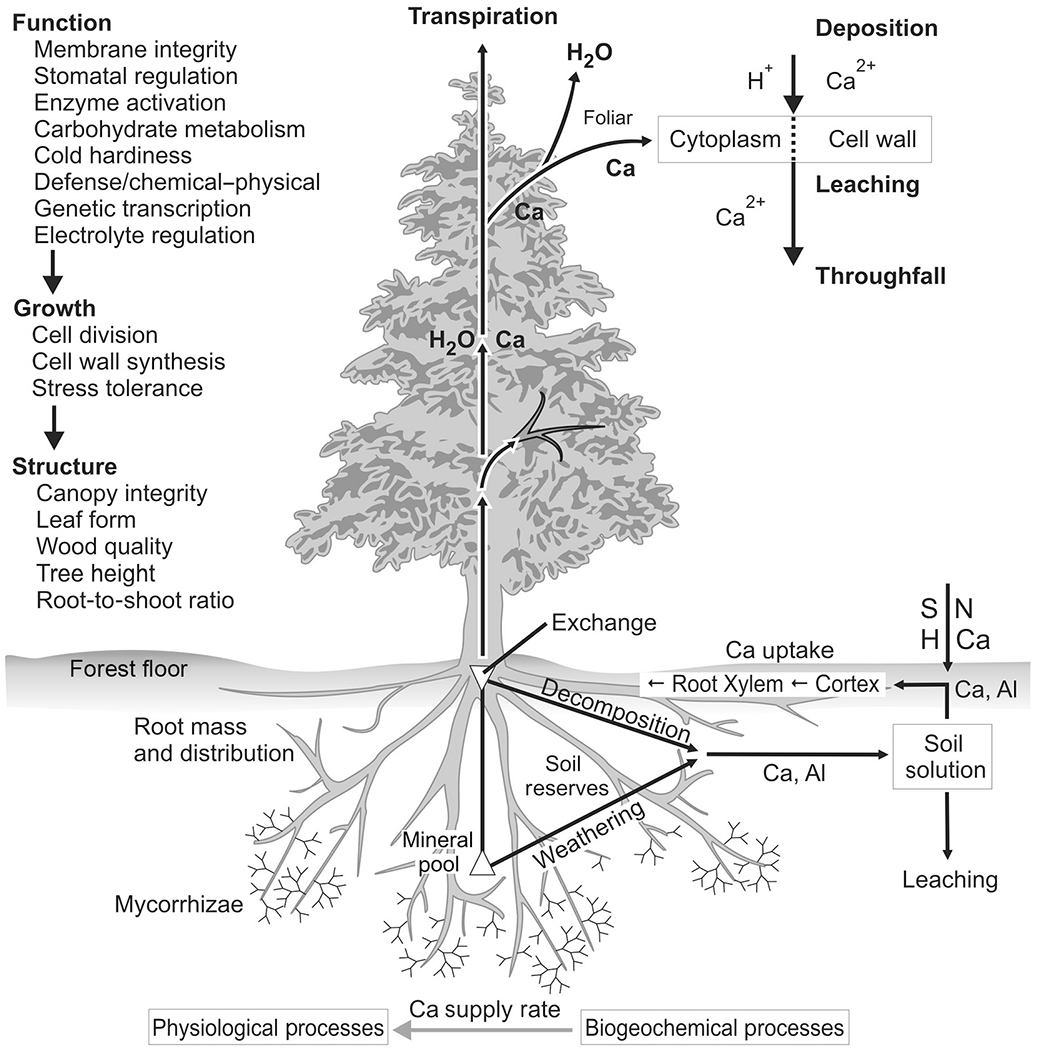
Conceptual diagram of how nitrogen deposition alters calcium cycling and dependent tree and ecosystem structure and function. Modified from Fenn et al. 2006.

Some of the best-understood and most persistent tissue and cellular mechanisms of N effects on trees occur via soils. Excess N deposition can acidify soil (Fig. 1.3.1) by accelerating the leaching of base cations, in particular Ca and Mg (see Fig. 2), major buffering agents to soil acidity. Historically, most Ca and Mg depletion (especially in the eastern United States) was attributable to S deposition, though in the past 10–15 yr, N has increasingly become an important acidifying agent as S pollution has decreased by more than 35% (Greaver et al. 2012, Lajtha and Jones 2013, Vet et al. 2014).

Acidic soil conditions mobilize potentially toxic cations (notably Al and manganese [Mn]), whose impact is first noted at the root level (roots have various mechanisms to exclude or sequester phytotoxic cations belowground as a protective mechanism [Cronan and Grigal 1995, Marschner 2012; Fig. 1.3.2]); though, this acidification also affects other parts of the tree, including foliage. As acid conditions persist and base cations leach away, Al and Mn availability and uptake increase and can accumulate in leaves, causing physiological dysfunction (St. Clair et al. 2008; Fig. 1.2.1). Al and Mn are strong binders to negatively charged structures in leaves and are considered functionally immobile in the phloem. Multiple mechanisms of Mn toxicity have been suggested, including that Mn toxicity occurs in leaf apoplasts where it impairs photosynthesis and growth through a combination of reduced carboxylation efficiency because Mn competes with Mg as a Rubisco activation factor or by disrupting the photosynthetic electron flow in chloroplasts (Fernando and Lynch 2015). Excess Mn also competes with the transport and metabolism of other cations, which induces nutrient deficiencies (Fernando and Lynch 2015). For example, increased Mn levels result in low Mg:Mn ratios that are associated with reduced chlorophyll concentrations, carboxylation efficiency, photosynthesis, and growth (St. Clair et al. 2008). Accordingly, physiological dysfunction for sensitive tree species (e.g., sugar maple [*Acer saccharum*]) has been associated with Mn accumulation (St. Clair and Lynch 2004, St. Clair et al. 2005). Overall, although Mn toxicity is an important contributor to tree decline at least for some species and locations, it does not appear to be as prevalent an issue as the more widely documented Ca and Al impacts.

Acidity associated with chronic N deposition can directly affect foliage and aboveground biomass, and acid-induced foliar Ca leaching and reduced Ca incorporation within leaves are particularly important (Fig. 2). Acid conditions also leach Mg and K, but these are better retained due to the possibility of resorption via the phloem. The particular relevance of Ca depletion to reduced tree health has been verified, in part, because Ca addition has been shown to reverse these declines (Green et al. 2013, Battles et al. 2014). Acid deposition reduces soil Ca, limits root Ca uptake, and leaches Ca associated with the plasma membranes of mesophyll cells or the membrane-associated Ca (mCa) from foliar cells (DeHayes et al. 1999, Schaberg et al. 2001, 2002, Borer et al. 2005; Fig. 1.2.1 and 1.2.4). Biologically available Ca is concentrated in cell walls and associated membranes, and this Ca is vulnerable to leaching loss through the cuticle and stomata. This Ca loss can alter the composition of the cuticle and destabilize membranes (Fig. 1.2.2). Acidity associated with additional N deposition has directly induced foliar Ca leaching in many north temperate forest tree species, including *Picea rubens* (red spruce), *Platismatia glauca* (white spruce), *A. saccharum* (sugar maple), *Acer rubrum* (red maple), *Betula alleghaniensis* (yellow birch), and *Pinus strobus* (eastern white pine) (Schaberg et al. 2001 and references therein, Pardo et al. 2011).

Reductions of mCa, both from foliar acid leaching (discussed previously) and via N-induced soil Ca loss (Dauer and Perakis 2014), destabilize cell membranes, decreasing the pool of messenger Ca that triggers biochemical stress response cascades. Reduced stress response predisposes trees to greater damage when exposed to internal and external stresses, and increases the likelihood of reduced tree growth and increased mortality (Schaberg et al. 2001 and references therein; Fig. 1.2.5). Movements of labile messenger Ca also act as important regulators of various aspects of C metabolism (McLaughlin and Wimmer 1999), so that limitations of this Ca may downregulate net C assimilation. Consistent with this possibility, low foliar Ca is associated with reduced photosynthetic capacity (e.g., Ellsworth and Liu 1994; Fig. 1.2.7), and mitigation of Ca limitation through Ca addition has been shown to increase the photosynthetic surface area, wood production, and aboveground net primary productivity of trees (Battles et al. 2014, Mainwaring et al. 2014; Fig. 1.2.8), in part because improved Ca nutrition may trigger a shift in C allocation from fine roots to aboveground biomass (Fahey et al. 2016).

Low mCa concentrations may also be mechanistically related to changes in respiration patterns for trees already suffering from other stressors (e.g., thin/rocky soils, drought; Schaberg et al. 1997, 2002). Increases in respiration have been documented in a variety of plant types and tissues and could also be attributed to the associated reductions in membrane integrity. Arguably, cells utilize more energy to maintain ionic partitioning when a lack of mCa hinders membrane structure and function (Schaberg et al. 2002 and references therein). Another study showed that differences in foliar nutrition and physiology were not related to the form of N (reduced or oxidized) or anion (chlorine or NO_3_^−^) applied, but depended on whether the impacts were direct or secondary to N deposition (Schaberg et al. 1997). Declines in mCa may be the cause of increasing respiration in trees, but how treatments of additional N perturb soil, root, and mycorrhizal processes, instigating foliar mCa deficiencies, dysfunction, and decline, remains a topic of further research (Schaberg et al. 2002).

Nitrogen deposition also affects amino acid production and utilization in trees, which can influence the abundance of polyamines and the amino acid arginine as bioindicators of tree stress (Minocha et al. 2014, Veresoglou et al. 2014, Pál et al. 2015; Fig. 1.2.6). Large and persistent changes in the biosynthesis of polyamines can force significant homeostatic changes in cellular metabolism that perturb the overall cellular balance between C and N (Minocha et al. 2014 and references therein). Modified metabolic homeostasis can maintain elevated polyamine levels, making them useful indicators of long-term forest stress, including Ca deficiencies (Minocha et al. 2014). For example, a study involving six red spruce stands in three northeastern U.S. states showed that the concentration of the polyamine putrescine in foliage could be used as a reliable indicator for early detection of stress from Ca deficiencies in trees before any visual signs of damage, and provided indications of species-specific sensitivities to soil Ca levels (Minocha et al. 2014).

The long-term consequences from impacts on foliar and soil Ca pools are not yet fully understood. Calcium disruptions and changes in Ca:Al ratios (Cronan and Grigal 1995, Magill et al. 2004) and other ion ratios (Schaberg et al. 2001) seem to be linked to reduced tree growth. However, while some studies have linked altered Ca: Al ratios in both soils and foliage to the reduced growth and vitality of trees (Schaberg et al. 2001 and references therein), other work has shed doubt on the relevance of this ratio and the basis for its use as a risk indicator (Larssen and Carmichael 2000 and references therein). Clarification of the mechanistic role of Ca:Al ratios to summarizing the health, productivity, and management of forests is another research gap that would be useful to address.

#### At the individual level.—

At the individual level, N deposition has three potential effects on trees: (1) elevated growth and increased biomass allocation aboveground as N is a limiting nutrient, (2) soil acidification and all the subsequent effects (i.e., reduced growth, changes in C sequestration and allocation), and (3) effects on biotic interactions (e.g., effects on mycorrhizae and predisposition to pests and pathogens).

Individual reports suggest mixed impacts of soil N increases on root morphology and lifespan (Peng et al. 2017), including evidence of biomass increases (Brunner and Godbold 2007) and reductions (Grulke et al. 1998, Nadelhoffer 2000), no impacts on lifespan (Guo et al. 2008a), likely increases in root turnover (Nadelhoffer 2000), and species-specific changes in root systems (King et al. 1997). On the whole, however, evidence suggests that N deposition can increase photosynthesis, the shoot:root biomass ratio of trees, and decrease the absolute root biomass (Grulke et al. 1998, Tateno et al. 2004; Fig. 1.2.8). These changes can increase susceptibility to wind throw damage, particularly when combined with stimulated shoot production, O_3_ damage, and/or in drought years (Grulke et al. 1998). This increase in aboveground biomass is also strongly influenced by biotic interactions, which are discussed in more depth below.

As discussed above, N deposition acidifies soils and leaches base cations, which causes Ca and/or Mg depletion that can reduce tree growth and increase mortality, particularly in naturally acidic soils (Driscoll et al. 2001, Schaberg et al. 2001; Fig. 1.3.3, 1.3.4, and 1.3.5). The effects of soil acidification on plant response are discussed in depth in the preceding section (At the tissue and cellular levels—physiological responses). Heterogeneity of soils or the potential of microsite effects could impact trees at the individual level even if the whole community does not experience significant negative effects, though such fine-scale information is scarce.

Another mechanism by which N deposition affects forests is by altering biotic interactions between host trees and symbionts, pathogens, and pests. Changes in the diversity and function of mycorrhizae in response to increased available N are perhaps among the most important mechanisms that contribute to N deposition effects on forest health (Lilleskov 2005). Mycorrhizal fungi are important symbiotic organisms that are associated with the health of most terrestrial plant species (Smith and Read 2008). Trees can form with ecto- and arbuscular mycorrhizal forms, whose physiology differs substantially. Increased N deposition can lead to a reduction in C allocation to tree roots, including a decrease in their carbohydrate content (Fig. 1.3.6). Because mycorrhizae depend on roots for soluble carbohydrates, N deposition and mycorrhizal physiology are inherently linked (Wallanda and Kottke 1998). NITREX (a consortium of N saturation experiments in Europe [Wright and Rasmussen 1998]) studies show that the development of the fruitbodies of ectomycorrhizal fungi is especially sensitive to increased N and that the species diversity of fruitbodies decreased 30–40% after 3.5–4.5 yr of N treatment in the plot at Gårdsjön from 1990 to 1995 (Boxman et al. 1998). However, due to the enhanced production of nitrophilic ectomycorrhizal species, total aboveground fruitbody production can increase even with the loss of species (Boxman et al. 1998, Lilleskov et al. 2002, Gilliam et al. 2011). It should be noted that these readily visible changes under N loading rarely capture the full extent of species loss that occurs belowground (Lilleskov et al. 2002, Sirajuddin 2009, Cox et al. 2010).

Mycorrhizae with different hyphae extents respond differently to increasing atmospheric N deposition. Mycorrhizal fungi with limited soil exploration ability have been shown to respond positively to increased N loads and lower pH, while mycorrhizal fungi with medium-distance soil exploration abilities consistently exhibit decreased production of mycorrhizal hyphae (Suz et al. 2014). NITREX studies also show that chronic N additions can decrease soil densities of both fine roots (Boxman et al. 1995, 1998) and mycorrhizal fungi (Boxman et al. 1998). This impact of N pollution is the reduced development of mycorrhizal mantle thickness and external mycelium along N gradients (Boxman et al. 1998). Among NITREX sites, the two with the highest ambient N deposition levels had considerably lower mycorrhizal root density (Boxman et al. 1998). The significance of thin-mantled mycorrhizal morphotypes for trees has not been examined, but it is expected that the absorption capacity of fine roots is reduced (Boxman et al. 1998). The absorption capacity of fine roots is one important pathway through which trees take up nutrients (Guo et al. 2008b), and so damage to the mycorrhizae that support this mechanism could be detrimental to tree nutrition.

#### At the community and ecosystem level.—

The response of forests to N deposition depends on which species are present, which in turn may impact tree diversity through differential tree growth and survival responses (Thomas et al. 2010). Tree nutrient-use strategies provide one way of assessing responses to added N, wherein trees with low nutrient-use efficiency (i.e., high N uptake per unit C capture) are thought to better exploit high N conditions than species adapted to low-N environments (Chapin et al. 1986). There also has been particular recent interest in understanding whether these responses may depend on the type of mycorrhizal associations, wherein trees with arbuscular mycorrhizae (e.g., *A. rubrum, A. saccharum, Fraxinus americana, Liriodendron tulipifera*, and *Prunus serotina*) that produce lower levels of proteolytic enzymes respond positively to increased N deposition when compared to ectomycorrhizal species (Thomas et al. 2010, Phillips et al. 2013; Fig. 1.3.7). Individual species departures from this pattern, however, suggest that combinations of traits and site-specific conditions may affect mycorrhizal responses to N deposition (Thomas et al. 2010). These responses may also be mediated by the ability of mycorrhizae to access other non-N nutrients in soil. While mycorrhizae (especially ectomycorrhizae) are clearly important agents of mineral weathering that can improve host plant nutrition, current evidence is equivocal as to whether mycorrhizal access to rock-derived nutrients is directly sensitive to host plant nutrient demands (Rosenstock 2009).

Community-level shifts in response to N deposition are often a product of species-specific functional differences. In both American and European studies, beech and oak were found to have no foliar response to varied N availability, while spruce, pine, and *Abies* (fir) species all responded negatively to N gradients (McNeil et al. 2007). Species-to-species differences correlated with two functional characteristics—leaf mass per area and shade tolerance—so that species with high leaf mass per area and high shade tolerance are less sensitive to elevated N deposition (McNeil et al. 2007). Among N-fixing trees that can convert atmospheric N_2_ gas into plant available forms, it is possible that species with actinorhizal N-fixing symbionts that do not downregulate fixation under high N supply may be less sensitive to N deposition than species with rhizobial associations that regulate N fixation rates (Menge et al. 2009).

Nitrogen- and S-related changes in other elements may also shape forest communities. For example, declines in Ca availability can reduce sugar maple seedling regeneration and survival, and lower sugar maple crown vitality (Juice et al. 2006, Halman et al. 2013, Sullivan et al. 2013, Talhelm et al. 2013). The near-complete dearth of sugar maple seedlings and saplings in acidified, base-poor hardwood forests in the Adirondack area could indicate a community shift toward other species (Sullivan et al. 2013). Indeed, some shifts have already been reported in Quebec, where acid-tolerant American beech (*Fagus grandifolia*) is expanding in forests once dominated by sugar maple (Duchesne et al. 2005). Evidence of the role of Ca in affecting seedlings of other species is lacking in the United States. However, some work has shown that with N amendments and other vegetative growth enhancements, the reproductive output of dominant oak species increases, but not the quality of the offspring (Callahan et al. 2008). Studies in the Hubbard Brook Experimental Forest in the White Mountains of New Hampshire have also shown an increase in aboveground net primary production and transpiration in response to watershed-level Ca addition (Green et al. 2013, Battles et al. 2014).

The ecosystem consequences of long-term N deposition and enrichment on trees can be particularly complex via interactions and feedbacks that involve other biogeochemical cycles. Phosphorus is often thought to limit plant growth when N is abundant (Vitousek et al. 2010). There is also some evidence that low N deposition may initially increase P availability, whereas high N deposition may lower P availability, changes that could result in complex yet currently unknown responses in forest composition (Crowley et al. 2012). There is clearer evidence for long-term effects of N deposition on Ca and other base cations in soils, a result of soil acidification and base cation leaching loss (Aber et al. 1998). While reducing N inputs can lower N availability to some degree, there is, however, less consistent evidence that soil buffering recovers to the same degree. Decadal-scale N fertilization experiments in Europe have shown that soil base status and pH may increase when N inputs are reduced (Högberg et al. 2006). However, reductions in acid deposition to northeastern U.S. forests that have increased soil O horizon pH and reduced exchangeable Al have not also consistently improved soil Ca availability (Lawrence et al. 2012, 2015). Studies along natural soil N gradients further raise the possibility that long-term soil N enrichment can cause persistently high N availability and N saturation (Perakis and Sinkhorn, 2012), ultimately depleting weathering sources of Ca and increasing forest reliance on atmospheric Ca inputs (Hynicka et al. 2016).

#### Interactions with other factors.—

Nitrogen deposition affects tree responses to stressors like drought, climate change, invasive species, biotic pests/pathogen, fire suppression, and other land-use history and management practices, and to other pollutants like O_3_ and S deposition (Fig. 1.4). In nearly all cases, the most severe impacts of N deposition occur in concert with exposure to multiple factors or stressors (Bal et al. 2015). For example, nutrient stresses increase the risk of injury or mortality from other biotic and abiotic stressors (Bal et al. 2015). If N deposition simultaneously increases aboveground biomass and water demand (Grulke et al. 2009), drought stress likely worsens. If O_3_ is also elevated, stomatal response is diminished, which likely increases vulnerabilities to drought stress.

Nitrogen addition is theoretically expected to exacerbate O_3_ sensitivity because N deposition leads to more leaf tissue, which is the sensitive organ to O_3_, but studies on herbs have not found much evidence for this (Bassin et al. 2007, 2009). The combined impacts of increased N deposition and O_3_ may cause increased physiological damage by reducing fine root biomass and carbohydrate allocation belowground (Fenn et al. 2003a, b). In addition to having fewer roots from O_3_ exposure, the benefits of N addition on root development was lost at higher O_3_ loads, and the impacts of O_3_ exposure increased as N deposition rose (Watanabe et al. 2012, Mills et al. 2016).

Some research has shown that trees, like lichens, exposed to increased N deposition have an elevated sensitivity to drought; though, the mechanism of effect differs. Because of higher N loads, possible mechanisms of effect for this increased sensitivity include larger leaf areas for potential water loss and a larger fraction of total biomass being aboveground. Another mechanism in winter drought could stem from soil freezing (Britton and Fisher 2007 and references therein). However, some studies have also shown that N addition increases drought tolerance (Villagra et al. 2013). This occurs by increasing water-use efficiency via higher efficiency of CO_2_ uptake with smaller stomatal openings. This nutrient water response appears to vary across species in a range of ecosystems (Hubbard et al. 2001, Buckley and Roberts 2006, Villagra et al. 2013).

Increased N deposition may also alter the susceptibility of trees to pests and pathogens, in part, because of the increase in the N content of foliage and the overall increase in foliage due to eutrophication. Combined exposures to insects, drought, and increasing N deposition can lead to tree mortality, even when N deposition levels are below calculated critical loads (McNulty and Boggs 2010). In apple orchards in the Okanagan Valley of British Columbia, Canada, an eight-year N addition study showed that *Phytophthora* crown and root rot significantly increased under this amendment (Utkhede and Smith 1995). Spatial analyses near poultry operations found that higher levels of N deposition produced by the poultry operations did not increase stand volume growth because gains were offset by elevated pitch canker-related mortality of slash pine trees (*Pinus elliottii* var. *elliottii* Engelm.) that correlated with increased N loads (Lopez-Zamora et al. 2007). The implications of this relationship between higher N loads and greater incidence of pathogens are that additional N may predispose trees to increased infection because plant tissues are fairly succulent (higher water content), which may facilitate fungus entry because a higher volume of water crosses cell membranes, bringing pathogens with it. The plants’ ability to inhibit pathogen development may also be reduced, and nutrient conditions are more favorable for the invading fungus (Lopez-Zamora et al. 2007). In conjunction with drought conditions, Jones et al. (2004) also suggest that high atmospheric N deposition increases tree susceptibility to bark beetle attack. Possible mechanisms include either decreasing resistance to other pests and pathogens or changing plant growth characteristics and nutrient quality. Another possible mechanism through which high N deposition increases tree susceptibility is direct acid damage to cuticular surfaces (Jones et al. 2004). High foliar N may also predispose *Pseudotsuga menziesii* (Douglasfir) to infection by Swiss needle cast, a native fungal pathogen (*Phaeocryptopus gaeumannii*) that decreases CO_2_ uptake and tree growth in forests across large areas of the Pacific Northwest (Waring et al. 2000, El-Hajj et al. 2004). Declines in foliar Ca associated with high N that reduce foliar membrane integrity may also increase susceptibility to Swiss needle cast (Perakis et al. 2006, 2013). Elevated N can also lead to greater production of N-rich defensive compounds, which can reduce tree vulnerability (Throop and Lerdau 2004).

Atmospheric CO_2_ concentrations and subsequent climate change also greatly influence the response of trees to N deposition. However, these interactions are not the focus of this paper and have been thoroughly addressed elsewhere (e.g., Reich et al. 2006, Arneth et al. 2010); thus, we only briefly discuss the main components below for this important modulating factor. As CO_2_ concentrations rise and climate changes, the interactions between C and N in terrestrial ecosystems will impact terrestrial productivity, future atmospheric CO_2_ concentrations, and climate feedbacks in complex ways (Luo et al. 2004, Reich et al. 2006). In addition to the interactions of the C cycle with N deposition and continued changes in interactions of N with other nutrients (Ca, Mg, P—discussed previously), there is emerging evidence that novel nutrient interactions, such as with K, for example (Sardans and Peñuelas 2015), may warrant further study. Climate change is expected to exacerbate many of the effects discussed above and mitigate others by influencing factors such as water availability, habitat changes, and increased levels of other pollutants. In European forests, direct tree damage due to water scarcity, hail damage, and uprooting by wind will likely be the most visual effect of climate change (Lamersdorf et al. 1998). Climate change may also increase forest fire risks and increase the frequency of pest infestations. Increased N deposition, both alone and when interacting with other factors such as climate change, elevated CO_2_, elevated O_3_ exposure, drought, and bark beetle outbreaks, can lead to many of the effects explained above (see Individual Responses). A small number of studies have looked at some of these interactive effects in small controlled plot studies (e.g., Zavaleta et al. 2003), but more work is needed to better understand these interactions and to understand for what species N deposition or climate change is more influential.

#### Research gaps.—

Research gaps (see Table 1), both methodological and content-based, continue to limit our understanding of the mechanisms through which N deposition affects trees. Here, we focus on those gaps that are critical to understanding the potential deleterious effects of N deposition on trees. Much of the body of literature on forest response studies and critical loads relies upon N fertilization treatments that aim to replicate N deposition but apply N only to the ground. Other gaps include the complexities of critical loads, the differential effects of reduced vs. oxidized N, and the potential reversibility of many of these effects for recovery to pre-industrial conditions, among others detailed below.

An important caveat to many experimental studies is that N fertilization treatments used in forest response studies and to establish critical loads are nearly always applied to the ground (as solution or N granules). This application of N to the ground does not replicate atmospheric deposition to tree canopies, especially in gaseous and particulate pollutant forms, and it is unknown how the effects of ground application of N differs from chronic atmospheric N pollutant fluxes to the canopy. Furthermore, differences in effects probably vary depending on local conditions. Canopy treatments may be essential to addressing this discrepancy and to more fully replicate mechanistically N deposition in the field (Nair et al. 2016). Even treating canopies with nutrient solutions does not approximate ambient longterm forest exposures to the multiple pollutant and nutrient compounds that occur in the atmosphere in a wide array of physical and chemical forms. Still, to the extent that the effects of N are primarily soil-mediated in some situations, N amendment treatments can be useful in simulating N deposition responses.

A study by Thomas et al. (2010) examined 23 individual tree species’ growth responses in the northeastern United States to increased N deposition, but for most other species (and especially those in the western United States), essential remaining questions include the following: What are the mechanisms resulting in widely varying responses to N among forest tree species? and what are the N critical loads for each species? Thomas et al.’s (2010) study also suggested that mycorrhizal type may be an important determinant of tree response to N. Phillips et al. (2013) suggested that this key finding deserves further study and incorporation into a framework to better understand nutrient dynamics and plant–soil interactions.

Little is known of the N critical loads at which trees become predisposed to pests and pathogens or increased drought stress nationally; though, some studies in California have shown that trees face increased mortality from bark beetles at critical loads at or above 39 kg N·ha^−1^·yr^−1^. However, this preliminary critical load value is likely an overestimate due to the low number of sites studied along the deposition gradient (Fenn et al. 2011). The difficulty in setting critical loads for such effects arises due to the involvement of multiple stressors, which are affected by climatic and other environmental conditions and by the inherently unpredictable and episodic nature of insect or disease outbreaks.

An associated research gap is the differential effects of reduced vs. oxidized forms of N on plants generally (Van den Berg et al. 2016); though, it is likely that these responses depend on soil pH and nitrification rates (Stevens et al. 2011). A better understanding of the mechanism and thresholds of response of mycorrhizal types to N deposition and the ecological significance of such responses would also be important. With so many confounding factors, it is often difficult to assign causality to specific individual mechanisms or stressors (McNulty et al. 2005).

Understanding how natural background N availability (and soil nutrient availability overall) predisposes forest responses to N deposition must be better evaluated. A case can be made that forests that are naturally N-enriched are acclimated to additional N, or near their tipping points of change, and that the response direction depends on the variable examined. We only have European NITREX studies to draw conclusions from regarding this topic, but those studies are confounded by specific historic land use and species differences among sites, so it is uncertain how generalizable these results are.

There is a need to better understand how other nutrient cycles equilibrate with increased N over time, particularly P and Ca, as these nutrients can become limiting under chronically high N supply despite vastly different biogeochemical response mechanisms (Vitousek et al. 2010, Perakis et al. 2013). Interactions among these elements also involve feedbacks with plant productivity and soil C cycling that ultimately determine whether and how N switches from a limiting nutrient to a cause of nutrient imbalances. With chronic N deposition, K and Mg may also become limiting; though, the extent of this phenomenon is unknown. Magnesium leaching is seen commonly in Europe with Norway spruce (*Picea abies*), but does not seem to be as important as Ca and Al in the United States. Magnesium limitations have only been sporadically noted in the United States for species like sugar maple (Schulze 1989, St. Clair et al. 2008).

The reversibility of soil acidification and recovery to pre-industrial conditions are also complicated because different compounds (in particular, SO_4_^2−^, N species, and H^+^) respond along different time scales. Thus, for slow-acting chemical processes like pH changes and chemical and biological processes in ecosystems, reversibility may occur very slowly or not at all in some regions (Beier et al. 2003), yet more quickly in others (Högberg et al. 2006). This, in turn, may depend on whether essential base cations to neutralize acidity originate from large weathering fluxes or from limited atmospheric inputs (Hynicka et al. 2016). Research gaps remain in our collective understanding of the mechanisms through which N deposition affects trees. For a summary of these research gaps and more beyond the narrative text provided here, please see Table 1.

## Conclusion

While N is an essential nutrient needed by all lichens and trees, our review focused on the deleterious effects of N, which suggests that environmental factors are important in modulating the response of epiphytic lichens to N deposition; a prominent example of this is their greater sensitivity to N deposition under arid conditions. The primary mechanisms by which N deposition affects trees in the United States also vary by region, primarily as a result of associated geologic and climatic differences. In the northeastern United States, where many soils are prone to acidification and low base saturation, Ca depletion and resulting physiological effects on trees are of considerable concern, although eutrophication effects also occur. In the more arid climates throughout much of the western United States, eutrophication effects on trees are prevalent and may depend on natural N levels in the soil.

The most damaging effects of N deposition to trees result from acidification and widespread cation imbalances. Interactions with other stressors such as freezing injury, drought, pest outbreaks, low nutrient supplies, and O_3_ also play a role. Nitrogen-fertilized plants are often predisposed to pests or diseases, and a similar phenomenon has been shown in lichens. Questions remain regarding the relative toxicities to lichens and trees of chronic N deposition in oxidized vs. reduced forms. This question is particularly relevant as the proportion of N deposition in reduced forms will likely continue to increase in the future (Du et al. 2014, Li et al. 2016b).

Large reductions in emissions of SO_x_ and NO_x_ in some areas of the United States may lead to at least partial ecosystem recovery from soil and surface water acidification over time. However, climate change looms large as another environmental factor expected to affect forest responses to N deposition in unknown ways over time and across regions. For example, increasing temperature and, in some instances, more frequent stagnant atmospheric conditions as a result of climate change are expected to result in increased O_3_ and particulate pollution exposure in some areas (Galloway et al. 2014). Under changing air pollution exposures such as these, N deposition exposures and fluxes are expected to change as well. Already, trans-Pacific transport of O_3_ from Asia to the western coast of the United States is increasing background O_3_ levels (Zhang et al. 2008). These and other environmental changes in the future will alter environmental conditions in forests and interact with N deposition, thus affecting the mechanisms of forest responses to N deposition.

## Figures and Tables

**Table 1.** Summary table of the prominent knowledge or data gaps regarding nitrogen deposition and mechanisms of impact to lichens and trees.

Research gaps regarding lichens	Research gaps regarding trees
How climate and human factors interact with N-induced mechanisms and other factors	The western U.S. and semiarid forests in general are understudied
Effects of N deposition on symbiotic relationships	Critical loads for individual tree species
Variation in N uptake among different species of photobionts	How chronic N deposition induces effects on trees (e.g., thresholds, mechanisms, and interactions with other stressors)
Molecular mechanisms for how lichens metabolize N	How do other nutrient cycles respond to N deposition to shape long-term effects?
Connections between metabolism and physiology of lichens and N tolerance	Are soil changes important more in terms of nutrients (Ca, Mg) or toxicity (Al, Mn)? In what geographic regions and why?
Interaction of N tolerance with light influx and humidity	How do background soil properties predispose trees to effects of N deposition? And how does this change through time?
Protective function of lichen secondary compounds and role in NH_4_^+^ and NO_3_^−^ uptake and tolerance	Some evidence that reducing N inputs lowers N availability, but less evidence that soil buffering also recovers The effects of N addition within canopies, as most studies are soil N additions
Pathways of transformation and assimilation of reduced and oxidized N	Relative impact of reduced vs. oxidized N for direct foliar effects and soil-mediated effects
Separation of NH_4_^+^ and pH effects	Better understanding of the mechanisms and thresholds of response of mycorrhizal types to N deposition and the ecological significance of such responses. Do mycorrhizal community changes in response to N affect tree vigor and forest sustainability?
Interactions of NH_4_^+^ adsorption with apoplastic pH and metal concentrations	More research on how reproduction and seedlings are affected for species other than sugar maple

*Note:* More details on selected topics are provided in Research Gaps sections for lichens and trees, respectively.
